# Supplementary figures and images for: A small protein encoded by a putative lncRNA regulates apoptosis and tumorigenicity in human colorectal cancer cells
Source: eLife. 2020 Oct 28;9:e53734. doi: 10.7554/eLife.53734 (PMC7673786; doi:10.7554/eLife.53734)

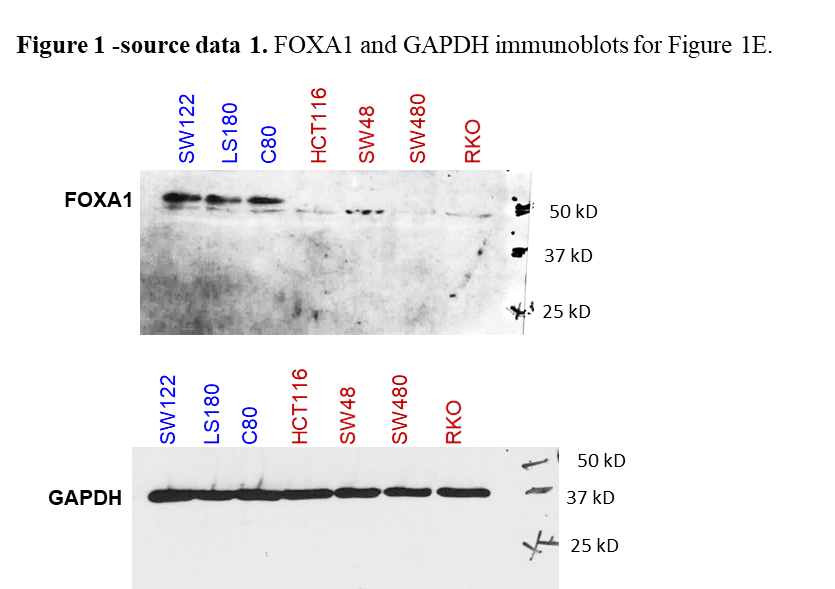

Supplement: Figure 1—source data 1. [file elife-53734-fig1-data1.docx]

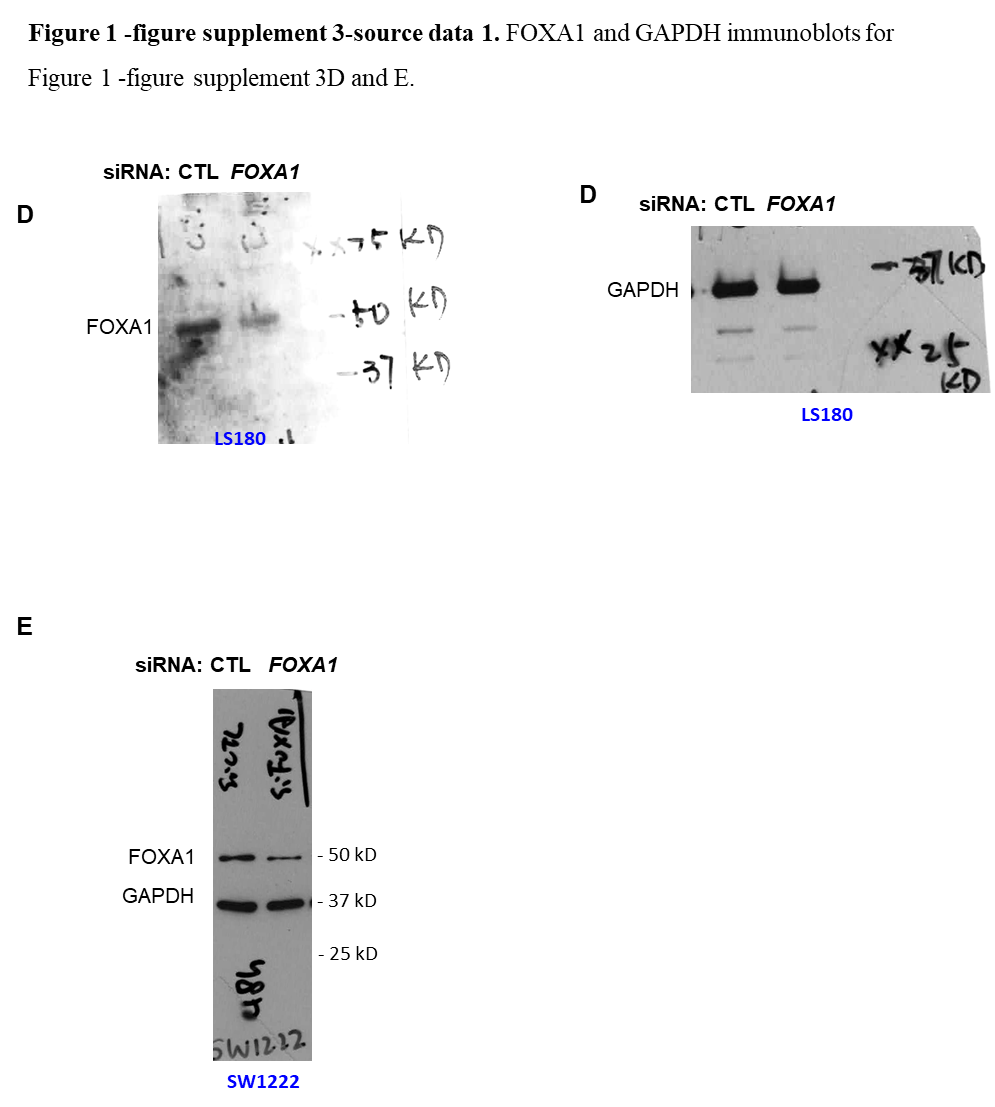

Supplement: Figure 1—figure supplement 3—source data 1. [file elife-53734-fig1-figsupp3-data1.docx]

**Figure 2-source data 1.** FORCP and Histone H3 immunoblots for Figure 2D.


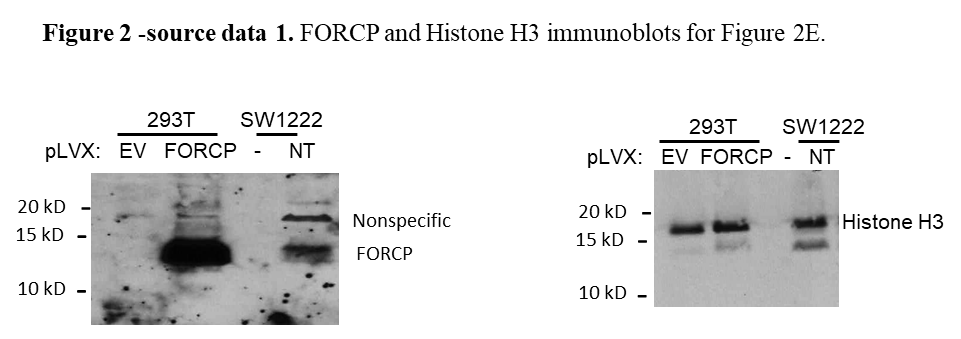

Supplement: Figure 2—source data 1. [file elife-53734-fig2-data1.docx]

**Figure 2 – source data 2.** FORCP and Histone H3 immunoblots for Figure 2E.


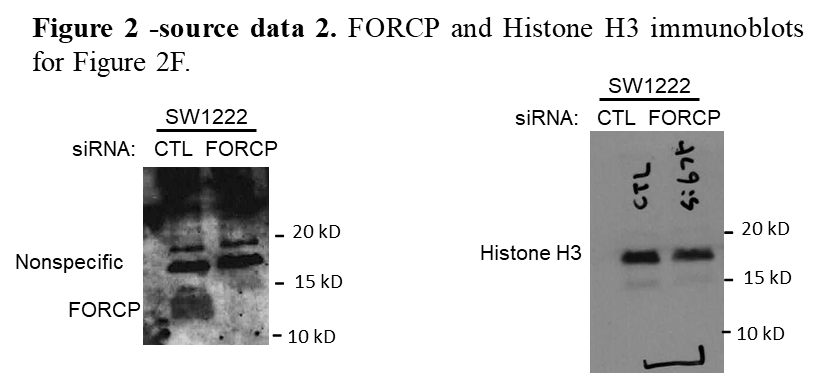

Supplement: Figure 2—source data 2. [file elife-53734-fig2-data2.docx]

**Figure 2-source data 3.** FORCP-FLAG and GAPDH immunoblots for Figure 2F.


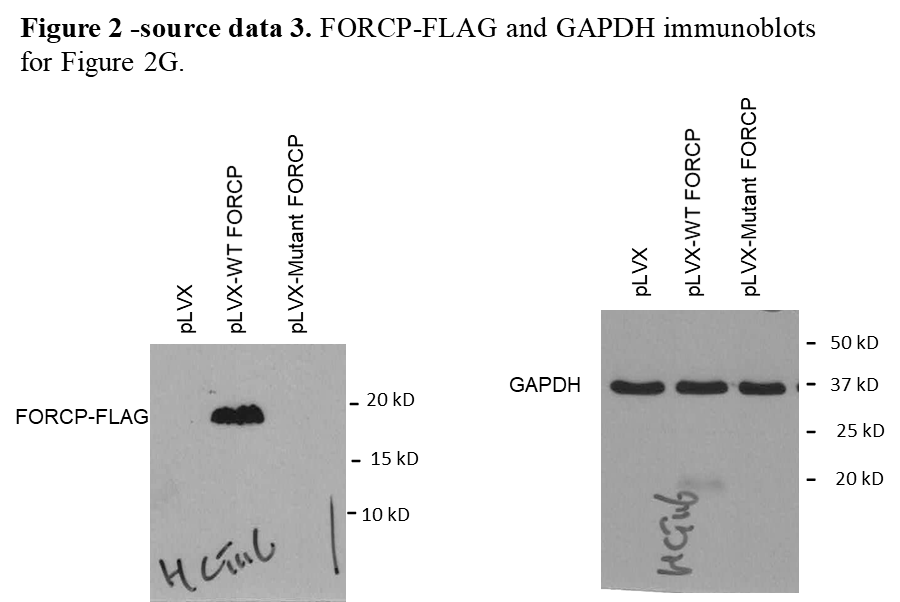

Supplement: Figure 2—source data 3. [file elife-53734-fig2-data3.docx]

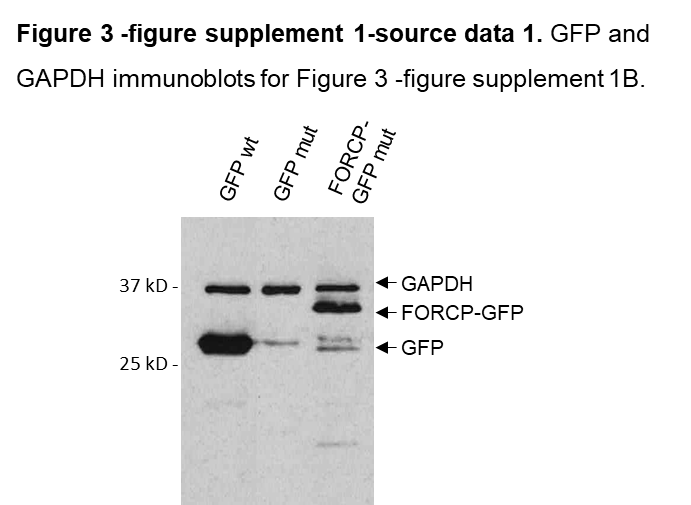

Supplement: Figure 3—figure supplement 1—source data 1. [file elife-53734-fig3-figsupp1-data1.docx]

**Figure 4-source data 1.** Cleaved caspase-3 and GAPDH immunoblots for Figure 4B.


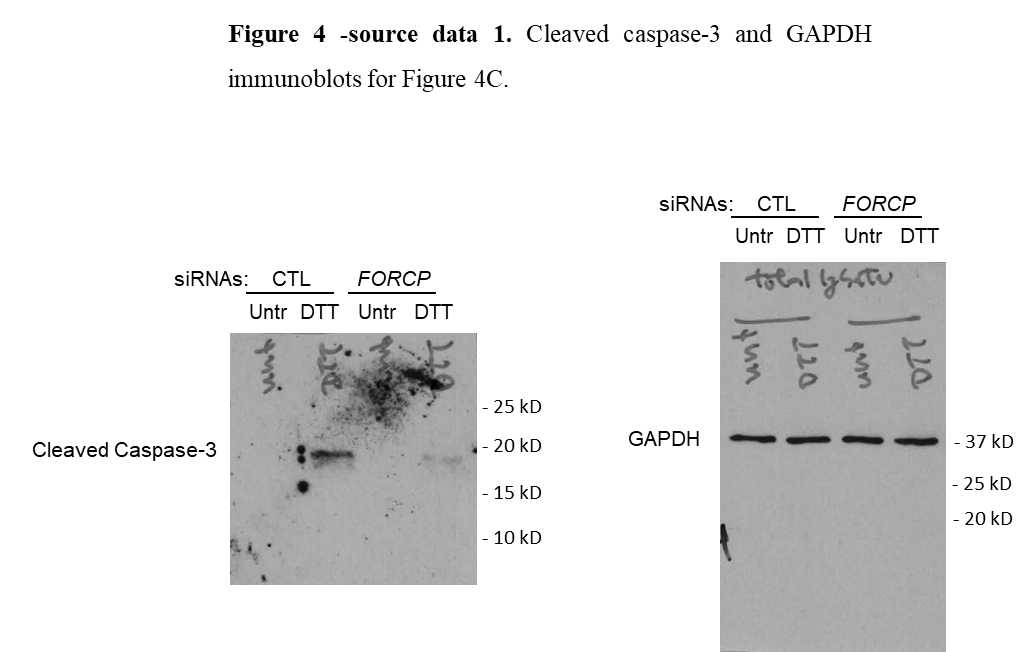

Supplement: Figure 4—source data 1. [file elife-53734-fig4-data1.docx]

**Figure 4-source data 2.** Cleaved caspase-3 and GAPDH immunoblots for Figure 4C.


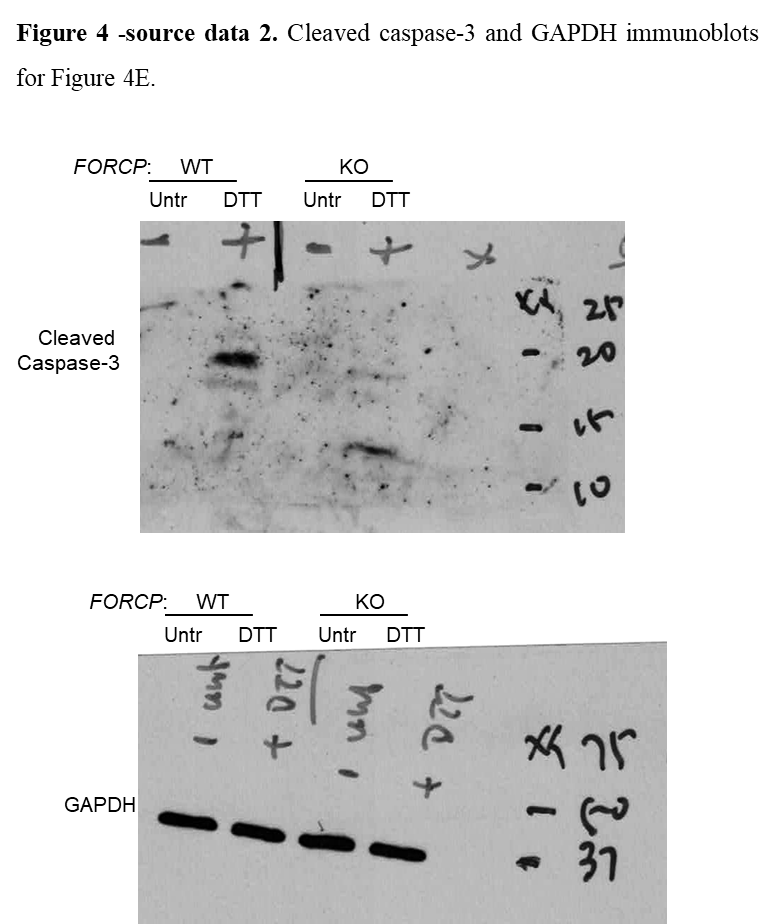

Supplement: Figure 4—source data 2. [file elife-53734-fig4-data2.docx]
